# Natural Products for Cancer Therapy: A Review of Their Mechanism of Actions and Toxicity in the Past Decade

**DOI:** 10.1155/2022/5794350

**Published:** 2022-03-11

**Authors:** Yasir Osman Ali Abdalla, Bavani Subramaniam, Shaik Nyamathulla, Noorasyikin Shamsuddin, Norhafiza M. Arshad, Kein Seong Mun, Khalijah Awang, Noor Hasima Nagoor

**Affiliations:** ^1^Institute of Biological Sciences (Genetics and Molecular Biology), Faculty of Science, Universiti Malaya, 50603 Kuala Lumpur, Malaysia; ^2^Department of Pharmaceutical Technology, Faculty of Pharmacy, Universiti Malaya, 50603 Kuala Lumpur, Malaysia; ^3^Centre for Natural Products and Drug Discovery (CENAR), Faculty of Science, Universiti Malaya, 50603 Kuala Lumpur, Malaysia; ^4^Department of Clinical Pharmacy and Pharmacy Practice, Faculty of Pharmacy, Universiti Malaya, 50603 Kuala Lumpur, Malaysia; ^5^Centre for Research in Biotechnology for Agriculture (CEBAR), Universiti Malaya, 50603 Kuala Lumpur, Malaysia; ^6^Department of Pathology, Faculty of Medicine, Universiti Malaya, 50603 Kuala Lumpur, Malaysia; ^7^Department of Chemistry, Faculty of Science, Universiti Malaya, 50603 Kuala Lumpur, Malaysia

## Abstract

The ethnopharmacological information gathered over many centuries and the presence of diverse metabolites have made the medicinal plants as the prime source of drugs. Despite the positive attributes of natural products, there are many questions pertaining to their mechanism of actions and molecular targets that impede their development as therapeutic agents. One of the major challenges in cancer research is the toxicity exerted by investigational agents towards the host. An understanding of their molecular targets, underlying mechanisms can reveal their anticancer efficacy, help in optimal therapeutic dose selection, to mitigate their side effects and toxicity towards the host. The purpose of this review is to collate details on natural products that are recently been investigated extensively in the past decade for their anticancer potential. Besides, critical analysis of their molecular targets and underlying mechanisms on multiple cancer cell lines, an in-depth probe of their toxicological screening on rodent models is outlined as well to observe the prevalence of their toxicity towards host. This review can provide valuable insights for researchers in developing methods, strategies during preclinical and clinical evaluation of anticancer candidates.

## 1. Introduction

Natural products, especially plant-based medicines and remedies have been studied since time immemorial due to their therapeutic effectiveness against various maladies and illnesses [1]. This prompted the exploration and isolation of numerous phytoconstituents with clinical benefits and these compounds have had profound impacts in medical science. For cancer therapy, up to 60% of anticancer candidates in clinical use that exhibited significant efficacy are natural product derivatives [2]. They serve as good sources of lead molecules and offer affordable materials in modern drug discovery. Plant-based natural products cause fewer undesirable side effects probably due to their similarity with chemical entities found in the human diet that have significant tolerance induction ability.

The secondary metabolites of plants such as flavonoids, tannins, alkaloids and terpenoids are well known to possess substantial anticancer capabilities [3]. They trigger, promote or modulate the metabolic pathways that can alter proliferation, migration and apoptosis in cancer cells via a multitude of biological mechanisms. Thus, not surprisingly phytoconstituents are the principal sources of chemotherapeutic drug investigations in preclinical and clinical studies of cancer. For instance, paclitaxel, a plant alkaloid was discovered in 1962 while screening natural products for treatment of cancer. It is commercially marketed in the name of Taxol® and has become one of the most effective drugs till date against breast and ovarian cancer [4].

The drug development process from its source to a finished product is an extremely costly and complex process that can take 12–15 years [5]. The toxicity of natural products and isolated compounds is a major concern in drug discovery and development [6,7]. Therefore, an in-depth investigation for safer natural drugs is always a prerequisite. Preclinical and clinical phases for the new chemical entities (NCEs) are important obligatory steps in drug development to assess the safety and efficacy of the test compound with an aim to predict any potential adverse events that may result after treatment. Toxicity tests are also imperative to identify the relationship between a dose, its potential side effects, anomalies on genetic material and on vital organs as well as to estimate toxicokinetic profiles. Besides, some of the major challenges encountered during drug development are low solubility, functional group reactivity, molecular complexity, and instability of investigational new drugs [8].

Therefore, the current review is an attempt to summarise a few plant extracts and phytoconstituents that are in the limelight in the past decade for significant anticancer activity. The underlying mechanisms and toxicities of these phytochemicals and plant extracts available in current literature were also summarised. A broader understanding of their mechanisms and limitations can benefit in the development of plant-based effective anticancer chemotherapeutic agents and help in the validation of their merits for clinical studies.

## 2. Anticancer Activities Reported on a Few Notable Plant Extracts of the Past Decade

**Figure 1:**
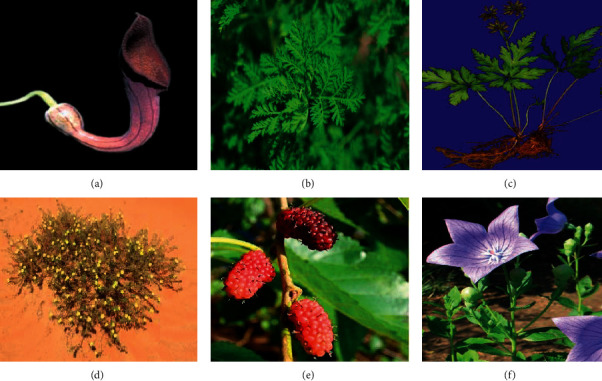
Plants under review with anticancer activities. (a) *Aristolochia baetica*, adopted with permission from [9], (b) *Artemisia annua*, adopted with permission from [10], (c) *Coptidis rhizome*, adopted with permission from [11], (d) *Fagonia indica*, adopted with permission from [12], (e) *Morus alba*, adopted with permission from [13], (f) *Platycodon grandifloras*, adopted with permission from [14].

The anticancer activity of plant extracts or their isolates that have been studied *in vitro* and *in vivo* against different cancer cell lines is presented in the following sections. These plants (Figure 1) and their mechanism of actions are presented in Table 1.

### 2.1. Aristolochia baetica (A.baetica)

*A. baetica* is a wild species from the *Aristolochiaceae* family that is distributed in the southern regions of Spain, Morocco, and Portugal. The plant grows in thickets, stream sides, and edges of woods. Historically, various plant parts were used by the Moroccans as treatments for several illnesses. For example, the roots were used for cancer and digestion-related illnesses while the flower and aerial parts were used for rheumatism and as abortifacient respectively [35,36]. Its anti-inflammatory and antiseptic properties made it as an excellent medicinal decoction used traditionally in several regions of Morocco ([15]). Many researchers reported that all parts of *A. baetica* can initiate toxic effects [37]. *A. baetica* contains a group of derivatives from aristolochic acids. Other than these derivatives include alkaloids, anthraquinones, phenolic compounds, steroids, and terpenes [38].

In cancer studies, hexane, chloroform, and ethyl acetate extracts of *A. baetica* inhibited proliferation of MCF-7 breast cancer cell lines with chloroform extracts being the most significant. The presence of aristolochic acid I in the extracts believed to be served as the predominant inhibitor of cancer cell proliferation [15]. Similar cytotoxic results against other cancer cell lines were observed due to aristolochic acid or related compounds from various species of *Aristolochia* genus [39]. In another study, the methanolic extracts of *A. baetica* roots suppressed proliferation of T-24 urinary bladder cancer, HT-29 colon cancer and HepG2 liver cancer cell lines [16].

### 2.2. *Artemisia annua* (*A. annua*)

*A. annua* belongs to *Asteraceae* family, is a type of short-day plant commonly found in the Asian region and commercially grown in the North American and African regions. It is referred as “Ginghao” in China, which is translated as sweet wormwood [40]. The plant itself has a brown, rigid stem and is resistant to predatory insects and pathogens [41]. Traditionally it is used in Chinese medicine, *A. annua* is prominently known for its antimalarial constituent, artemisinin. Artemisinin and its derivative are effective against *Plasmodium falciparum* that causes cerebral malaria and has been approved as the first-line treatment choice for malaria [42].

In recent years, *A. annua* extracts and artemisinin have been studied for their anticancer properties [43]. *A. annua* extract is endowed with anti-inflammatory activities [44]. As for artemisinin, it was found to have an effect in the reduction of TNF-*α* gene expression. The semisynthetic derivative of artemisinin, artesunate was tested on a multiplicity of cancer cell lines but was observed to be most effective against leukemia and colon cancers [45]. Although artemisinin is the dominant compound with therapeutic effects, it is not the most effective anticancer agent of this plant [46,47]. Instead, the plant consists of various biologically active substances that exert anticancer effects when present in combination [47]. Hence, extracts of *A. annua* have been investigated for their anticancer effects.

The ethanol/water (80/20%) extracts of *A. annua* stem and leaves were tested on HeLa and AGS human gastric cell lines. The leaves extract had a higher phenolic acid content and was more effective in inhibiting the growth of both cell lines at 500 mg/mL therapeutic concentration [17]. The *A. annua* extract with acetonitrile maceration were also shown to be cytotoxic towards MDA-MB-231 (breast), MCF-7 (breast), MIA-PaCa-2 (pancreas), PC-3 (prostate) and A549 (lung) cancer cell lines. In addition, the extract demonstrated antitumour and antiapoptotic effects towards TNBC MDA-MB-231 xenografts grown on chick chorioallantoic membrane (CAM) and in nude mice [18].

### 2.3. *Coptidis rhizome(Syn: C. rhizoma*)

*C. rhizoma* is the dried rhizome of *Coptis chinensis* Franch., *Coptis teeta* Wall., *Coptis deltoidea* C. Y. Cheng et Hsiao, *Coptis japonica* Makino or *Coptis japonica* var. *dissecta* of the *Ranunculaceae* family [48]. It is referred to as ‘Huanglian' in Chinese medicine and is widely found and consumed in Asian countries. Historically, *C. rhizoma* has been used as an anti-inflammatory traditional Chinese medicine to eliminate heat, remove dampness, and for detoxification. It was during the Han Dynasty that *C. rhizoma* was listed in the Shennong's classic Materia Medica for its medicinal property [49].

Over the years, *C. rhizoma* has been used to treat several illnesses including diabetes, eczema, diarrhoea, jaundice and high fever. Its many pharmacological properties such as antiviral, antibacterial, antifungal, antidiabetic, antioxidant, anti-inflammatory, and anticancer effects, make it one of the most versatile therapeutic natural products [50–52]. *C. rhizoma* constitutes about 120 chemical components ranging from alkaloids, volatile oils, organic acids, lignans, and flavones [53,54].

In hepatocellular carcinoma treatment, *C. rhizoma* extracts were shown to downregulate VEGF via inactivation of eukaryotic elongation factor 2 (EEF2) in HepG2 and MHCC97-L cells, to suppress angiogenesis [19]. It also demonstrated inhibition of the Rho/ROCK signalling pathway to prevent migration of MHCC97-L cells [20]. In addition, *C. rhizoma* exhibited anticachectic effects on oesophageal cancer by downregulating tumour IL-6 production in YES-2 cells and xenografts in a mice model [21]. The extract was also able to inhibit cell growth and induce apoptosis in Hep3B cells through increased expression of Bcl-2 and activated caspases [55].

### 2.4. *Fagonia indica (F. indica)*

*F. indica*, is a flowering plant from the family *Zygophyllaceae,* also referred to as “Dhamasa” [56]. The plants of the *Fagonia* genus are commonly found in Middle-eastern countries and have been utilized traditionally for several ailments such as colds, cough, digestive problems, asthma and dysentery [57,58]. These plants are versatile pharmacologically with anti-inflammatory, antioxidant, antimicrobial, antidepressant, antiviral, and hepatoprotective properties as they are rich with phytochemicals such as flavonoids, saponins, alkaloids, amino acids, and terpenoids [59].

In cancer studies, *F. indica* aqueous extracts demonstrated anticancer efficacy against many cancers. *F. indica* phytoconstituents have promising cytotoxic properties to destroy cancer cells by blocking the mutant epidermal growth factor, tyrosine [60]. The steroidal saponin glycosides extracted from the aerial parts of *F. indica* induced apoptosis-mediated cell death against MDA-MB-468 breast cancer and Caco-2 colon cancer cells. In MCF-7 breast cancer cell line, the compound demonstrated significant necrotic activity through cell lysis [22]. In addition, the aqueous extract *F. indica* was tested against wild-type and chemotherapy-resistant RKO and H630 human colon cancer cells. The results showed that the extract prevented proliferation and migration of the cancer cells and induced cell death via Akt/MAPK signalling pathway. A reduction in VEGF, NF-ĸB and ICAM-1 expressions were also observed. In another study, indicacin isolated from the methanolic extract of *F. indica* was found to be 51.40% cytotoxic at 6.25 mM/mL dose against H-29 human colorectal cancer cell line [23].

### 2.5. *Morus alba* L. *(M. alba)*

*M. alba*, is a moderate-sized white mulberry that belongs to the *Moraceae* family, and is native to India, China and Japan. It is commonly cultivated worldwide where silkworm is raised, whereby its leaf serves as the main source of nutrient for silkworms. The plant is referred to as Tutam (Sanskrit), Tut (Hindi), Malbari (Malayalam), and Musukette (Tamil) (*The Wealth of India: A Dictionary of Indian Raw Materials and Industrial Products (Industrial Products—Part I)* 1949); [61]. Traditionally, its extracts are used for cough, insomnia, asthma, wound healing, diabetes and edema treatments. *M. alba*'s phytoconstituents include phytosterols, saponins, triterpenes, flavonoids and others as active components. The leaves are sources of quercetin, 1-deoxynojirimycin, apigenin and rutin while the root is a source for polysaccharides [61].

*M. alba* functions as an antidiabetic, antistress, antimicrobial, antioxidative, antihelminthic, and anticancer natural product. In cancer therapy, *M. alba* root bark extract-initiated cell growth arrest and apoptosis in SW480, human colorectal cancer cell line. In this study, the extract demonstrated ROS and GSK3*β*-dependent-ATF3 activation and cyclin D1 proteasomal degradation [24]. In addition, methanolic extract of *M. alba* leaves inhibited proliferation of Calu-6 (pulmonary), HCT-116 (colon) and MCF-7 (breast) cancer cell lines [25]. Another study conducted on HepG2 human hepatoma cell line showed that the methanolic leaves extract arrested cell cycle in G2/M phase to induce apoptosis and prevent proliferation of the cells [26].

As for the phytoconstituents isolated from *M. alba*, albanol A is isolated from the root extract. It induced apoptosis and cytotoxicity towards HL-60 human leukemia cell line. Its mechanism of action included topoisomerase II activation, reduction of procaspases 3, 8, and 9, and increase in Bax/Bcl-2 ratio to stimulate cell death [27]. Besides that, lectin isolated from *M. alba* leaf extracts were observed to have antiproliferative effects on MCF-7 human breast cancer cell line. In HCT-15 human colorectal cancer cell line, lectin promoted cell cycle arrest and cytotoxicity. Lectin's proapoptotic effects were attributed to its ability to activate and release caspase-3 [28].

### 2.6. *Platycodon grandifloras* (*P. grandifloras*)

*P. grandiflorus*, also known as the Chinese bellflower, or balloon flower, is a type of perennial herb under the *Campanulaceae* family. It is widely distributed in Northeast Asia and has been used in food and traditional medicine in China, Japan and Korea [62]. The first record of the plant was found in Shennong Bencao in China before being documented in the Japanese Hanaoka Seishu [63]. The plant species is rich in many nutrients, making it suitable to be processed as food items such as pickles and salads [52]. In addition, *P. grandiflorus* was traditionally used to treat phlegm, cough, sore throat and other illnesses [64]. Its many active phytoconstituents that range from flavonoids, phenolics, saponins, polysaccharide and anthocyanins responsible for important anti-inflammatory, immune-stimulatory, hepatoprotective and anticancer properties.

In cancer therapy, *P. grandiflorus* induced apoptosis by reducing Bcl-2 expression, increasing Bax expression, and activating caspase and mitochondrial cytochrome c release in SKOV-3 human ovarian cancer cells [29]. It also demonstrated dose-dependent downregulation of MMP-9 and MMP-2, thus suppressing viability and invasion of HT-1080 fibrosarcoma cell line [30]. Furthermore, *P. grandiflorus* polysaccharides had significant effects in the inhibition of U14 cervical tumour growth in mice models. The polysaccharides induced apoptosis, increased P19ARF and Bax protein expressions, and decreased mutant p53 protein expression [31].

Platycodin D, a major triterpenoid saponin extracted from *P. grandiflorus* roots, have also been extensively studied for its pharmacological activities such as anti-tumor, anti-inflammatory, anti-obesity, and antiallergy [62,65]. In MCF-7 breast cancer cell line, platycodin D activated caspases and PRP cleavage, thus causing cytotoxicity in a dose- and time-dependent manner [32]). In human leukemic U937 cells, platycodin D activated Egr-1 gene for the eventual production of ROS that stimulated apoptosis and cell death [33]). In another study, Platycodin D was found to inhibit telomerase activity via downregulation of human telomerase reverse transcriptase (hTERT) expression and inducing cytotoxic effects on leukemic cells [34]).

## 3. Anticancer Activities Reported on a Few Promising Phytoconstituents

**Figure 2:**
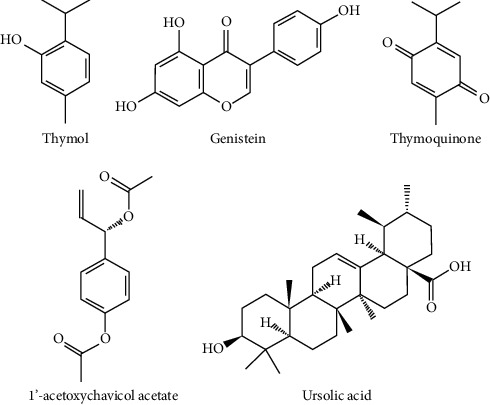
Chemical structures of phytoconstituents with anticancer activities (Source: PubChem).

In the past decade preclinical studies on few phytochemicals attracted many researchers and hence discussed in this review. The efficacies of some of these compounds are detailed below and their chemical structures are given in Figure 2.

### 3.1. 1′-Acetoxychavicol Acetate

1′-Acetoxychavicol acetate (ACA) is a type of hydrophobic ester isolated from *Alpinia conchigera* and *Alpinia galanga* of the *Zingiberaceae* family. The rhizomes of the plant are used predominantly in local cuisines of South-East Asia and are well known for their benefits in alleviating respiratory and gastrointestinal ailments [66]. They are herbaceous, perennial plants that contain various metabolites in their rhizomes. The nonpolar extracts of the rhizomes reported many types of plant sterols such as stigmasterol, *β*-sitosterol and several closely related phenylpropanoids such as ACA and 1′-acetoxyeugenol acetate (AEA). ACA being the major phytoconstituent of the plant, has been widely investigated for its pharmacological activities which include antiallergy, anti-inflammatory, antiulcer, antiviral and antitumour [67–69].

The most studied pharmacological activity of ACA was its ability to exhibit antitumour activity (Table 2). Apoptosis is caused by ACA via inhibition of the NF-*κ*B signalling pathway, activation of caspase 3 and expression of apoptosis-inducing ligand and death receptor. Initiation of apoptosis occurs *via* the mitochondria and Fas-dependent pathways [84]. Other studies suggest ACA induced apoptosis by increasing caspase-3, and DNA fragmentation with cell cycle arrest [70,71]. ACA also alleviated bone-related complications by causing osteoclastogenesis *via* blocking of RANKL-induced NF-*κ*B activation and IFN-*β* mRNA expression in cancer-associated bone loss [72,85]. It has also been reported that ACA induces autophagy via Beclin-1-independent pathway in an *in vitro* study [81]. In addition, downregulation of proinflammatory cyclooxygenase-2 (COX-2) and cyclin D1 expression in tumours were observed, which was further potentiated when coadministered with cisplatin [83].

ACA in combination therapies such as with, recombinant human alpha-fetoprotein (rhAFP); and *Mycobacterium indicus pranii* (MIP) and cisplatin (CDDP); has exhibited significant synergistic effects against A549 human lung cancer, PC-3 human prostate cancer and 4T1 mouse mammary cell lines [74,80] via NF-ĸB inactivation. Furthermore, ACA-loaded nanostructured lipid carriers for targeted therapy to CXCR4-expressing PC-3 cancer cells, demonstrated significant cytotoxicity, antimigration and anti-invasion properties *in vitro* [74]. Moreover, Bharadwaj and coworkers reported inhibition of colorectal cancer cell proliferation by ACA in SW480 cell line via cell cycle arrest, upregulation of p21 expression, significant reduction of cyclin D and genetic material impairment [78].

### 3.2. Genistein

One of the most studied preclinical anticancer phytoconstituent is genistein, chemically known as 4′,5,7-trihydroxyisoflavone. It is a type of isoflavone that is naturally present in soybeans and soy products. Isolation of genistein was first conducted in 1899 from a flowering species, *Genista tinctoria* of the *Fabaceae* family [86]. This was followed by successful discoveries of the compound in other plants such as soybeans, fava beans, and lupin [87]. Synthetic genistein was produced in the year 1928 [88], and have since been extensively studied for its anticancer properties (Table 3).

Genistein has been shown to induce apoptosis via the activation of caspase-9 and caspase-3 in HeLa human cervical cancer cell line [92], inhibition of the NF-ĸB pathway and regulation of caspase-3 and p38 MAPK signalling pathway in HT-29 human colon cancer cell line [93,94]. It has also been found to induce cell cycle arrest at the G_2_/M phase in HGC-27 (human gastric carcinoma), MDA-MB-231 (human breast cancer), HCT-116 and SW-480 (human colon cancer) cell lines. The mechanism of cell cycle arrest was mediated by Ras/MAPK/activator protein-1 for MDA-MB-231 and ATM/p53 and p21Waf1/Cip1 for the colon cancer cell lines [53,91,96]

Besides, the antiangiogenesis effects of genistein have been demonstrated in the downregulation of an angiogenic protein, vascular endothelial growth factor (VEGF), in human bladder cancer cells, oral squamous cell carcinoma, and thyroid cancer cells [90,99,100]. Moreover, genistein have been shown to have significant antimetastatic properties against salivary adenoid cystic carcinoma cells (ACC), lung cancer cells (A549) and colon cancer cells (HCT116) via inhibition of several metastatic gene expressions including MMP-2 and MMP-9 [89,95,97]. Genistein treatment also downregulated the expression of an epithelial-to-mesenchymal transition (EMT) transcription factor, in melanoma cells (B16F10) [98].

### 3.3. Thymol

Thymol is a type of phenol that is obtained from thyme oil and is chemically known as 2- isopropyl-5-methylphenol. It is a colourless natural monoterpene found predominantly in thyme species such as *Thymus vulgaris* and *Thymus zygis* [101,102]. Although thyme has been used as an antidote and an ingredient in medicinal concoctions and ointments for centuries, thymol was only first isolated in 1719 by Caspar Neumann [103]. In the late nineteenth century, thymol was further discovered to have therapeutic effects against the hookworm epidemic [104]. Since then, thymol has been exploited for its beneficial pharmacological properties such as antioxidant, anti-inflammatory, antibacterial, antifungal and anticancer activities [101].

In terms of anticancer efficacy (Table 4), thymol's cytotoxicity was observed on several cancer cell lines such as MCF-7 (breast), THP-1 (leukemia), P388 (leukemia), MG63 (osteocarcoma) and Hep-2 (laryngeal carcinoma) [105,107,108,112]. Specifically, apoptosis-mediated cell deaths were noticed in human glioblastoma cells through Annexin V/P1 staining [106]. In leukemia, thymol upregulated Bcl-2 associated X protein (Bax) expression and downregulated B-cell lymphoma (Bcl-2) expression, to promote apoptosis in HL-60 cells [109]. In addition, thymol resulted in mitochondrial-regulated apoptosis in MG63 cells [112]. Necrosis was also stimulated by thymol in human glioblastoma and Hep-2 cells [106,113]. Furthermore, cell cycle arrest in G0/G1 phase was promoted by thymol in MCF-7, CEM (human T-cell leukemia), P815 (mastocytoma) and K-562 (myelogenous leukemia) cell lines [110,111].

### 3.4. Thymoquinone

Thymoquinone, also known as 2-methyl-5-isopropyl-1, 4-benzoquinone, is a type of monoterpene molecule extracted from the *Nigella sativa* L. seed of the *Ranunculaceae* family. The seed itself has a historical reputation for treatments of various diseases in many middle eastern and far eastern countries. Its many bioactive constituents including thymoquinone, p-cymene and *α*-pinene, are responsible for its anti-inflammatory, antimicrobial, antioxidant, antiasthmatic, antihypertensive, and anticancer properties [114, 115]. Thymoquinone, the major essential oil constituent of the *N. sativa* seed, was first extracted in 1963 [116]. In addition to its many pharmacological activities, it has been found to exhibit significant anticancer effects specifically by initiating the production of reactive oxygen species (ROS) in many different cancer cell lines [117] (Table 5).

In breast cancer studies, thymoquinone could prevent proliferation of cancer cells by inducing p38 phosphorylation via activation of ROS generation, suppressing tumour growth *in vivo*, downregulating the expression of antiapoptotic genes such as, XIAP, survivin, Bcl-xL and Bcl-2, inhibiting production of Ki-67 tumour aggressor, and upregulating the level of catalase, superoxide dismutase and glutathione [119]. Thymoquinone also demonstrated JNK phosphorylation in human colon cancer cells and squamous cell carcinoma [123,126,127] and reduction of ERK phosphorylation in glioblastoma and lung cancer cells [124,125].

Thymoquinone's attenuation of the PI3K/Akt signalling pathway to inhibit cell growth, proliferation, and angiogenesis is observed in HTB-9 bladder cancer; MDA-MB-468 and T47D breast cancer and; TFK-1 and HuCCT1 cholangio-carcinoma [118,120,122]. Inactivation of the NF-ĸB pathway was also observed in mouse cancer cells, TFK-1 and HuCCT1 cholangio-carcinoma, HEPG2 hepatic carcinoma and KBM-5 myeloid leukemia [122,128–130]. Moreover, thymoquinone reduces the expression of TWIST1 transcription factor to reduce invasion and metastasis of BT549 human breast cancer cell line [121].

### 3.5. Ursolic Acid

Ursolic acid (UA), chemically known as 3-*β*-hydroxy-urs-12-en-28-oic acid, is a type of pentacyclic triterpenoid, isolated from a variety of medicinal plants such as *Origanum vulgare* (oregano) leaves*, Lavandula angustifolia* (lavender), *Eucalyptus* (eucalyptus) leaves and *Malus domestica* (orchard apple). The molecular weight of ursolic acid is 456.7 g/mol and its melting point ranges between 283 and 285°C. As a hydrophobic compound, it is soluble in organic solvents such as acetone, methanol and pyridine but insoluble in water. Ursolic acid's pharmacological functions include anti-inflammatory, antidiabetic, antioxidative, antihyperlipidemic, and anticancer activities [131].

In terms of its anticancer properties, ursolic acid has been extensively studied on breast cancer cell lines (Table 6). In MCF-7 and MDA-MB-231 human breast cancer cell lines, ursolic acid has been found to downregulate STAT3, EFGR and cyclin D1 to arrest cell cycle, induce apoptosis and prevent cell proliferation [132]. The compound also inhibits migration and invasion of MDA-MB-231 cells by controlling the c-Jun N-terminal kinase (JNK), protein kinase B (Akt) and mammalian target of rapamycin (mTOR) signalling pathways [133]. *In vivo* studies of ursolic acid on mice induced with MMTV-Wnt-1 breast tumour cells also demonstrated modulation of the Akt/mTOR signalling pathway, induction of apoptosis and cell cycle arrest to reduce tumour volume [134].

On SGC7901 gastric and HepG2 hepatic cancer cell lines, ursolic acid downregulated the expression of COX-2 to induce cytotoxicity, apoptosis and prevent proliferation of cancer cells [64,140]. Apart from its proapoptotic function in these cells, ursolic acid promoted mitochondrial dysfunction via activation of caspases 3, 8, and 9, and downregulation of Bcl-2 expression [82,137,138,141]. Besides, ursolic acid reduced matrix metallopeptidase 9 (MMP-9) expressions to inhibit metastasis of HeLa, HT1080 (fibrosarcoma) and C6 (glioma) cells [135,136,139].

## 4. Toxicity Details of the Selected Anticancer Plant Extracts

The toxicity of plant extracts described in this review has been presented in the following sections.  Table 7 is a summary of the toxicity of these plant extracts on animal models, it details the type of assessment, route of administration, pathological changes during the study.

### 4.1. Aristolochia baetica

*A. baetica* aqueous extract was screened for safety on Swiss albino mice model. The acute toxicity test showed no mortalities or signs of toxicity when administered orally. *A. baetica* aqueous extract accelerated the rate of mice run by about 3 to 5 minutes. In another observation, the aqueous extract with a dose of 4 g/kg produced a shortness of breath, abnormal locomotion, and 16% of deaths. In sub-acute toxicity study, no clinical signs were observed. As for the liver markers, *A. baetica* increased the level of AST when mice were given 2 g/kg/day dose. An evaluation of the renal markers showed that the creatinine concentration was increased in group (1.5 g/kg/day) compared to the control group. Kidney histopathological examinations showed no changes but, when mice were treated with 1.5 and 2 g/kg/day doses, renal necrosis, inflammatory infiltrate, cortical necrosis, and tubular degeneration were recorded [147].

### 4.2. Artemisia annua

Swiss albino mice model was selected for safety screening of the hydro-ethanolic plant extract of *A. annua*. The extract was administered orally with 5000 mg/kg as the highest dose. There was no lethality or toxic reactions found at any of the doses of *A. annua* extract. The absence of toxicity symptoms suggests that *Artemisia annua* was nontoxic and was well tolerated [142].

### 4.3. Coptidis rhizoma

The acute toxicity test was conducted in mice model which was administered with *C. rhizoma* extract. The results showed no toxicity related signs during the 14-day acute study. The LD_50_ was established at dose higher than 7000 mg/kg of the body weight. A longer toxicity study for 90 days was conducted in Sprague-Dawley rats. There were no side effects or clinical signs on survival that could be attributed to the administration of the extract. There were no behavioural changes, no abnormalities in body weights, food and water consumptions for treated rats compared to the control group. When haematology parameters were analysed, the outcome showed that haemoglobin, red blood cell count, white blood cell count, lymph leukocyte count, mononuclear leukocyte count and granular leukocyte count were not affected significantly by the dosages of extract. The biochemical parameters indicated that there was a significant increase in the ALT and AST at dose of 3.76 g/kg. In the histopathological examination, the dose of 3.76 g/kg caused degeneration of hepatocytes and aggregation of inflammatory cells in the lung. In a subchronic toxicity study, the NOAEL of *C. rhizoma* extract was at 1.88 g/kg [143].

### 4.4. Fagonia indica

The 14-day acute toxicity study of *F. indica* was conducted on male albino mice at 5 mg/kg of extract and 10 mg/kg doses. At the end of the study, the results showed no morbidity or behavioral changes in the treated groups. The plant extracts did not cause significant changes on the level of ALT and AST but a significant reduction in ALP level was recorded when animals were administered with the ethanolic extract of the plant. This result indicated that no possible cholestasis occurred at the dose levels tested. Histologically, the results revealed that the hepatocytes were not affected by the plant extract [145].

### 4.5. *Morus alba*

In the acute toxicity test, the ethanolic extract of *M. alba* was administered orally to female Swiss mice. The extract showed low level of toxicity in mice and death was detected at a dose of 2000 mg/kg. MCV was reduced and serum alkaline phosphatase was increased in animals that received the highest dose. A reduction in leukocytes counts was observed at 300 and 2000 mg/kg doses [144].

### 4.6. *Platycodon grandiflorus*

In subchronic toxicity study of *P. grandiflorus*, no significant differences were observed. Clinical signs, body weight, food and water consumption, ophthalmic examination, urinalysis, haematology, serum biochemistry, necropsy findings, and organ weights were relatively normal under the treatments. However, serum creatinine was increased significantly in treated group compared to control. In addition, the organ weight values did not differ significantly between groups. Histopathological examination showed centrilobular hepatocellular hypertrophy in the liver of some rats treated with extract. NOAEL of this study was established at a dose of more than 3000 mg/kg/day in rats [65].

## 5. Preclinical Toxicological Screenings of Anticancer Phytoconstituents

### 5.1. 1′-Acetoxychavicol Acetate

Acute toxicity and 28 day subacute toxicity studies conducted using ACA on Sprague- Dawley rats showed that ACA's NOAEL was 2.22 mg/kg. Exposure to parenteral doses between 0.66 and 6.66 mg/kg neither caused fatality nor body weight loss and morphological changes during acute and subacute studies. Further there was no significant impact of ACA on either organ weights or relative organ weights compared to control throughout subacute study and recovery period. The haematopoietic and biochemical assessments showed a significant reduction in WBC in acute studies at middle and high dose treatment, however, these parameters were within the normal range in the subacute study. In terms of hepatotoxicity, ACA showed mild lobular hepatitis in healthy nontumour bearing SD rats. Also, the treated groups did not show impaired glomerular filtration or nephrotoxicity as seen by normal urine output and components compared to normal untreated rats. Other parameters such as electrolytes and BUN levels are important parameters used to assess renal function and these exhibited no significant changes thus indicating no nephrotoxicity. In the histopathological analysis microscopic sections of vital organs were taken to look for abnormalities and pathological manifestations. In kidneys, both glomeruli and tubules appeared normal with mild interstitial nephritis. In lung sections pneumonitis of different intensity in all treated rats was observed. However untreated rats also exhibited similar manifestations [148].

### 5.2. Genistein

Genistein had a low order of toxicity in acute toxicity study and was well-tolerated in repeated dose toxicity study. There was an increase in food consumption and subsequently an increase in body weights of rats in acute and subchronic toxicity studies. Hematological examinations showed reduction in RBCs when rats were treated with high doses. In terms of biochemical parameters, at high dose, a slight increase in gamma glutamyl transferase in male and female rats was observed. Male rats' organs such kidney, spleen, adrenal and testes weights were increased while for females, increased weights of liver, kidney, spleen, ovary and uterus were observed. A majority of the results in these studies were limited to 500 mg/kg/day (high dose) and were reversible. The NOAEL of genistein was established at 50 mg/kg/day [149].

In another acute toxicity study of genistein in mice, alanine aminotransferase (ALT), aspartate aminotransferase (AST), and alkaline phosphatase (ALP) levels were elevated, and degenerated liver tissue was prominent in 500 and 1000 mg/kg genistein treated groups. Elevated serum ALT, AST, and ALP levels in these animals suggest hepatotoxicity [150].

### 5.3. Thymoquinone

Acute toxicity study was conducted for thymoquinone in rats with no significant changes in behavioural appearance and morbidity as a result. Also, there were no significant changes in the body weight, food intake, organ-to-body weight ratio, and haematological, biochemical and histopathological profile with all parameters within the normal range. There were no significant differences (*p* > 0.05) in the serum ALP, ALT, creatinine, urea, total protein, albumin and total bilirubin levels [151].

In another study, after acute oral administration, the LD_50_ value of thymoquinone was found at 2.4 g/kg. Hypoactivity and difficulty in respiration were observed in animals treated with the highest doses of thymoquinone. The results indicate that acute oral toxicity of thymoquinone in mice is low and the compound is well tolerated [152].

### 5.4. Thymol

In acute and subacute toxicity studies of thymol, the haematological and biochemical parameters were not altered. However, the histopathological examinations of the organs exhibited changes in the lung with no other changes in the rest of the organs. The body weight deviated only in male rats that were given 500 mg/kg dose of thymol. The relative weight of the organs did not differ significantly. NOAEL was established at a dose greater than 250 mg/kg/day, and the essential oil of *Thymus vulgaris* was shown to cause moderate oral toxicity [146].

### 5.5. Ursolic Acid

In chronic oral toxicity study of UA, the results showed that UA did not cause death, abnormal body weights or abnormal pathology at tested doses. Additionally, no other toxicological changes in terms of behaviour, neurotoxicity, coagulation, haematology or clinical was observed postadministration of UA. Thus, oral dosing of UA for 90 consecutive days is not toxic at any of the doses. The NOAEL for UA was established at a dose higher than 1000 mg/kg/day [153].

In a recent toxicity study conducted by Mishra and coworkers, UA was reported to cause elevation in neutrophils, urea in blood, and ALP enzymes. On the contrary, a low level of some other haematological parameters such as platelets and lymphocytes were revealed by the subacute toxicity study of UA. In histological examinations, UA showed recoverable alterations in some major organs, especially in liver, spleen, and kidney. Hence, UA might cause mild toxic side effects when used for a prolonged period [154].

## 6. Discussion

Plants and their secondary metabolites have a major stake in drug discovery and medicine including cancer research. The purpose of this review article is to identify the plant extracts and metabolites in recent times with significant preclinical anticancer reports. The review also attempts to introduce and describe ACA, a molecule which was extensively studied in our labs. An overview of anticancer molecular mechanisms of these test compounds will provide an in-depth understanding of the compound's safety for precise medication and to generate minimal toxicity in clinical use.

The plant extracts studied in this review were aqueous and organic extracts of *A. baetica, M. alba, F. indica*, *P. grandiflorus, C. rhizoma* and *A. annua* that were found to induce apoptosis and necrosis, and exhibit antiproliferation, antimigration and anti-invasion activities on several cancer cell lines both *in vitro* and *in vivo*. The versatility of *C. rhizoma* extracts in downregulating VEGF for anti-antiogenesis, inactivating Rho/ROCK signalling pathway for antimigration and upregulating Bcl-2 and caspase activity of antiapoptosis, should be noted [19,20,55]. As for *F. indica*, the extracts were demonstrated to inactivate the Akt/MAPK signalling pathway and reduce tumorigenic factors such as VEGF, NF-ĸB and ICAM-1 [23]. Besides that, *M. alba* extract induced ATF3 activation, cyclin D1 proteasomal degradation and cell cycle arrests [24,26]. *P. grandiflorus* on the other hand, could reduce Bcl-2, increase Bax, activate caspase, induce mitochondrial cytochrome c release, and downregulate MMP-9, MMP-2, and mutant p53 proteins [29–31].

Based on these different mechanisms of action it can be understood that the plant extracts offer significant positive attributes against various cancer cell types. However, there are some limitations to the use of plant extracts for clinical studies. For instance, the diverse metabolite profile caused by the extraction procedures, the efficacy and properties of the same species grown in different environments vary due to their distinct profile of medicinal compounds [155]. The influence of environmental factors on the composition of secondary metabolites in natural products is undeniable and well documented in the literature. In addition, geographical location, soil quality, extraction method and genotype of the plants can also cause inconsistencies in herbs. Therefore, the heterogeneity of natural products is one of the major challenges that limits the reproducibility of therapeutic outcome. Nevertheless, proper standardization of plants and optimization of the extraction procedures could offer a solution that can avoid variations. To obtain reproducibility in natural products' research a proper identification, authenticity of the plant species, its genus, variety and detection of markers is crucial. Macroscopical characters such as the shape, size, venation patterns of leaf, floral arrangements, inflorescence, type of fruit and microscopical characters such as type of stomata, vascular arrangements, trichomes, lignified tissues and cellular inclusions of the selected plants prior to extraction could offer insights on the authenticity and quality. Further, advanced analytical techniques like HPLC, LCMS or HPTLC could be valuable to detect single or multiple markers of the extracts which are directly connected to the therapeutic outcome. A study conducted by Sandikapura et al. [156] revealed that method of extraction can cause significant alterations in the secondary metabolite composition in the extracts which can seriously alter pharmacological effects. In addition, there is a need to emphasize uniformity in cultivation of these plants for therapeutic purposes by standardization of their chemical profiles via biotechnological and genetic studies [157].

On the contrary, certain metabolites extracted and isolated from plants display specific bioactivities that boost therapeutic effectiveness when used in isolation [158]. A recently studied preclinical phytoconstituent, ACA, is found to induce apoptosis via inactivation of the NF-ĸB pathway, mitochondrial and Fas-dependent dual pathways and activation of caspase-3 [84]. Besides ACA, genistein is an isoflavone that also initiates apoptosis by activating caspase-9 and caspase-3 [92]. Furthermore, thymol is a type of phenol that induces both apoptosis and necrosis on human glioblastoma cells [106]. In addition to inducing cytotoxic effects on many cancerous cells, thymol increases production of ROS that leads to cell membrane disintegration and DNA damage [159]. Thymoquinone induces p38 phosphorylation via activation of ROS generation and attenuates PI3K/Akt signalling pathway in breast and bladder cancers [118,119]. Ursolic acid too, can induce apoptosis, preventing cell proliferation and inhibiting migration and invasion of breast cancer cells [132,133].

Despite abundant plant extracts and metabolites identified as effective therapeutic agents with established mechanisms of action, further preclinical studies and safety assessments are required to provide information on their safety and efficacy for regulatory approvals. Hence, in early drug development, a crucial step is the assessment of toxicity profile via short and long-term studies in animal models with the selection of a clinically appropriate route of drug administration [148].

Firstly, observation of behavioral changes is useful in predicting toxicity at early stages. For instance, the correlation of food consumption and body weight can be evaluated to address the toxic effect. All the plant extracts in this review did not cause mortality when administered orally in animal models. In addition, these extracts caused no changes in food/water consumptions and body weights except for alteration in body weights for male animals treated with the essential oil extract of *Thymus vulgaris.* As for genistein, it led to a decrease in food consumption and body weight at higher doses. Other compounds caused no alterations during their experiments. The alteration in body weight is normally an indication of toxic effects caused by the investigational agent [148]. Similarly, in a clinical trial study of this compound on breast cancer, the patients demonstrated differences in body weight measurements because of the treatment. These clinically adverse events occur due to variations in metabolism and food intake, besides reduction in energy expenditure and physical activity [160].

Secondly, assessment of haematological parameters is crucial in toxicity studies because many anticancer drugs affect the bone marrow and subsequently cause alteration in blood production. Among the above plant extracts, only the extract of *M. alba* leaves resulted in reduction of MCV, MCHC and leukocytes levels. In terms of the phytoconstituents, a high dose of genistein treatment decreased the RBCs count and increased the reticulocytes. In addition, ursolic acid caused an increase in platelet count as with many other studies that demonstrated an elevation of platelet count, neutrophil count, and urea concentration with ursolic acid treatment [153]. It is well known that the cytotoxic effects of some plant extracts and compound fractions can lead to alteration in the production of blood cells and result in the suppression of immune system. Many plant extracts and metabolites have been reported to act directly on erythrocytes. In general, the extracts and metabolites cause reduction in haemoglobin which may imply their effect on hematopoiesis. These plant extracts may act directly on erythrocytes leading to a reduction in haemoglobin, and sometimes go further by destroying the cells [144].

Besides the above, *A. Baetica* extracts led to an increase in creatinine concentration [16]. Similarly, thymoquinone treatment caused an increase in urea and creatinine concentration. Increased creatinine concentration is associated with the decrease in kidney functions and damage progression. Creatinine level is also affected by muscle mass loss and drugs. However, this measure is not directly related to toxicity and is primarily a measure of the glomerular function of kidney [65]. In short, elevation in urea and creatinine levels can indicate nephropathy [161].

Further, biochemical parameters are important in assessing the toxicity profile of new chemical entities. Parameters that assess hepatocellular injury are liver transaminases, such as ALT, AST and ALP. Other parameters that are often evaluated to provide an assessment of liver function include albumin and clotting factors. Oral administration of genistein, thymoquinone, and extracts of *A. Baetica, F. indica,* and *C. rhizoma* caused an elevation of ALT and AST liver enzyme when the animals were treated separately. The opposite occurred when animals were treated with *M. alba* leaf extracts (reduction in ALT). But treatment of ACA resulted in a significant increase in total protein, albumin and globulin. Also, genistein at higher dose produced a slight increase in gamma glutamyl transferase. In liver, genistein induced the expression and activity of the ATP binding cassette transporter P-glycoprotein. The compound activates estrogen receptors *α* and *β* due to its structural similarity to 17-*β*-estradiol [162]. Acute liver injury is normally manifested with alanine aminotransferase (ALT), to be highly elevated at more than three times the baseline level.

Biochemical parameters that should be considered when assessing the renal function are the level of serum electrolytes, creatinine and blood urea nitrogen. The most commonly used urinary parameters to estimate the glomerular function is serum creatinine. Thymoquinone and the extract of *P. grandifloras* caused an increase in serum creatinine level. However, serum creatinine should not be the sole basis of estimation of renal function as there are many other factors that can affect the creatinine level, such as age, sex, muscle mass and low-protein diets. Other than the creatinine level, the blood urea nitrogen level is often measured concomitantly. Simultaneous increase of blood urea nitrogen level with an increase of serum creatinine may imply acute renal injury [163].

Lastly, assessment of histopathological changes is vital to see whether there is a damage caused by the anticancer agent. Hence, a comprehensive analysis must be done during toxicity studies. Most of the plant and compounds discussed in the review caused a change in the liver (Table 7 and 8). Extract of *C. rhizoma* caused degeneration of hepatocytes in the liver and aggregation of inflammatory cells in the lung. The reasons for the damages that occurred in the liver include destruction of hepatocellular function and release of liver enzymes as discussed earlier. Besides that, *A. baetica* was reported to cause renal necrosis. This was due to immunomodulatory properties of the extract which might be able to trigger an autoimmune response in the toxic lesions [147]. *Thymus vulgaris* L. essential oil showed a moderate inflammatory infiltrate in lungs and mild acute inflammation in the stomach. ACA causes inhibition of the inflammatory NF-*κ*B pathway [80,83]. ACA also enhanced levels of inflammatory cytokines (IL-6, IL-1*β* and TNF-*α*) which is confirmed by the induction of lung inflammation in animal models [73].

## 7. Conclusion

In the present review, a summary of some plant extracts and a few phytochemicals used for preclinical studies on cancer in the past decade have been discussed. The use of these natural products to target specific biological pathways to induce antitumour efficacies have been described to provide a detailed review on their specificity and molecular targets. The limitations about natural products are mainly due to the heterogeneity of the extracts. Plant extracts contain many active metabolites such as alkaloids, flavonoids, terpenes, saponins, steroids, glycosides and the mechanisms, therapeutic response is the combined effect resulting after synergistic, antagonistic and neutralization of their individual effects, to establish and identify the minimum effective dose of a given sample and its maximum tolerable dose toxicity studies are imperative. Preliminary preclinical toxicity study in animal models is an important regulatory requirement in drug development to assess the safety of the test sample prior to clinical evaluation. At later stages, these toxicity studies can be narrowed down at the fractions/compounds level aiming to push the potential therapeutic agent or candidate forward in the drug development process. Preclinical drug safety studies are essential at the early stages of development to avoid complications in later phases. Nevertheless, a comprehensive process of isolation, testing and toxicological evaluation of anticancer agents are important to achieve drug development, some of the major challenges encountered in the development of anticancer agents include lack of sufficient studies on efficacy, safety, solubility, stability, targeting, and toxicity profile.

## Figures and Tables

**Table 1:** The summary of anticancer effects of plant extracts and their isolates under review.

Plant	Extract/isolate	Cancer type	Cell line	Effects	Reference
*Aristolochia baetica*	Hexane, chloroform, ethyl acetate extracts	Breast	MCF-7	Antiproliferation	[15]
	Methanol extract	Urinary bladder	T-24	Antiproliferation	[16]
		Colon	HT-29		
		Liver	HepG2		

*Artemisia annua*	Ethanol/water extract	Gastric	HeLa	Inhibition of cell growth	[17]
			AGS		
				
	Extract of and acetonitrile maceration	Breast	MDA-MB-231	Cytotoxicity, antitumour, antiapoptotic *in vivo*	[18]
			MCF-7	Cytotoxicity	
		Pancreas	MIA-PaCa-2	Cytotoxicity	
		Prostate	PC-3	Cytotoxicity	
		Lung	A549	Cytotoxicity	

*Coptidis rhizoma*	Aqueous extract	Hepatocellular carcinoma	HepG2 MHCC97-L	Inactivation of EEF2, downregulation of VEGF, suppression of angiogenesis	[19]
			MHCC97-L	Inhibition of Rho/ROCK signalling pathway, antimigration	[20]
	Extract powder	Esophageal	YES-2	Downregulating tumour IL-6, anticachectic	[21]

*Fagonia indica*	Steroidal saponin glycoside	Breast	MDA-MB-68	PARP cleavage, caspase-3 cleavage, DNA fragmentation, apoptosis	[22]
			MCF-7	Cell lysis, necrosis	
	Aqueous extract	Colon	Caco-2	PARP cleavage, caspase-3 cleavage, DNA fragmentation, apoptosis	
	Indicacin	Colorectal	H-29	Cytotoxicity	[23]

*Morus alba*	Methanol extract	Colorectal	SW80	ROS and GSK3*β*-dependent-ATF3 activation, cyclin D1 proteasomal degradation	[24]
	Methanol extract	Pulmonary	Calu-6	Antiproliferation	[25]
		Colon	HCT-116		
		Breast	MCF-7		
	Methanol extract	Hepatoma	HepG2	Cell cycle arrest at G2/M phase, antiproliferation, apoptosis	[26]
	Albanol A	Leukemia	HL-60	Topoisomerase II activation, reduction of procaspases 3,8, and 9, increase in Bax/Bcl-2 ratio, apoptosis	[27]
	Lectin	Breast	MCF-7	Antiproliferation	[28]
		Colorectal	HCT-15	Cell cycle arrest, cytotoxicity	

*Plactycodon grandiflorus*	Water/ethanol extract	Ovarian	SKOV-3	Downregulation of Bcl-2, upregulation of Bax, activation of caspase and mitochondrial cytochrome c release	[29]
	Saponin	Fibrosarcoma	HT-1080	Downregulation of MMP-9 and MMP-2, cytotoxicity, antiinvasion	[30]
	Polysaccharides	Cervical	U14	Apoptosis, upregulation of P19ARF and Bax, reduction of mutant p53 protein	[31]
	Platycodin D	Breast	MCF-7	Activation of caspases, PRP cleavage, cytotoxicity	[32]
		Leukemia	U937	Activation of Egr-1 gene, production of ROS, apoptosis	[33]
			U937 THP-1 K562	Antiproliferation, downregulation of hTERT, inhibition of telomerase activity	[34]
				Antiproliferation	
				Antiproliferation	

**Table 2:** The anticancer effects of 1′-acetoxychavicol acetate on different cell lines.

Cancer type	Cell line	Effects	Reference
Breast	MCF-7	Cytotoxicity	[70]
		Activation of caspase-3, apoptosis	[71]
		Inactivation of NF-ĸB pathway and downregulation of its genes	[72]
	MDA-MB-231	Cytotoxicity	[73]
		Activation of caspase-3	[71]
	4T1	Downregulation of MMP-9 and VEGF, upregulation of IL-2 and IL-10, antitumour	[74]

Cervical	CaSki	Upregulation of has-miR-138, has-miR-210, and has-miR-744	[75]
	CaSki and SiHa	Overexpression of RSU1, antiproliferation, apoptosis	[76]
		Downregulation of miR-210, upregulation of SMAD4	[77]

Colon	SW-480	Cell cycle arrest, upregulation of p21, reduction of cyclin D, antiproliferation	[78]
	Colo 320	Antiproliferation, apoptosis	[79]

Lung	A549	Inactivation of NF-ĸB pathway and downregulation of its genes, cytotoxicity, antitumour	[80]
		Induced prosurvival autophagy through Beclin-1-independent pathway	[81]

Prostate	PC-3	Inactivation of NF-ĸB pathway and downregulation of its genes, cytotoxicity, antitumour	[62]
		Downregulation of CXCR4, VEGF, p65, and Ki-67, antitumour	[74]
		Antiproliferation, antimigration, antiadhesion, downregulation of Src, CD31, VEGF	[82]

Oral	HSC-4	Apoptosis, antimigration, antitumour, downregulation FasL, Bim, NF-*κ*B, COX-2 and cyclin D1	[83]

**Table 3:** The anticancer effects of genistein on different cell lines.

Cancer type	Cell line	Effects	Reference
Adenoid cystic	ACC	Inhibition of MMP-9, antimetastatic	[89]
Bladder	RT4, J82, 5637, T24	Downregulation of VEGF, upregulation PAL-1, endostatin, angiostatin, and THBS-1	[90]
Breast	MDA-MB-231	Activation of ERK1/2 pathway, induction of cell cycle arrest at the G2/M phase, downregulation of Cdk1, cyclin B1, and Cdc25 C	[91]
Cervical	HeLa	Activation of caspase-9 and -3	[92]
Colon	HT-29	Inhibition of NF-ĸB	[93]
		Activation of caspase-3 and p38/MAPK	[94]
	HCT-116	Anti-roliferation, cell cycle arrest at the G2/M phase	(Mizushina et al., 2013)
		Inhibition of MMP-9, COX-2, Ang-1, VASP, VEGF, anti-etastatic	[95]
	SW-480	Cell cycle arrest at the G2/M phase, apoptosis via ATM/p53 pathway	[53]

Gastric	HGC-27	Induction of cell cycle arrest at the G2/M phase	[96]
Lung	A549	Reduction of ERK1/2, PI3K/Akt and MMP-2	[97]
Melanoma	B16F10	Downregulation of Snail	[98]
Oral squamous	HSC-3	Downregulation of VEGF, anti-invasion	[99]
Thyroid	CAL-62, ACC 448, CGTH-W1, ACC 360	Cytotoxicity, downregulation of VEGF, hTERT, NF-ĸB genes, upregulation of PTEN and p21 mRNA	[100]

**Table 4:** The anticancer effects of thymol on different cell lines.

Cancer type	Cell line	Effects	Reference
Breast	MCF-7	Cytotoxicity, cell cycle arrest in G0/G1 phase	[105]
Glioblastoma	DBTRG-05 MG	Apoptosis, necrosis	[106]
Leukemia	THP-1	Cytotoxicity	[107]
	P388	Cytotoxicity	[108]
	HL-60	Upregulation of Bax, downregulation of Bcl-2, apoptosis	[109]
	K562	Cell cycle arrest in G0/G1 phase, reduction of DNA damage	[110]
	CEM	Cell cycle arrest in G0/G1 phase	[111]

Mastocytoma	P815	Cell cycle arrest in G0/G1 phase	[111]
Osteosarcoma	MG63	Cytotoxicity, mitochondrial-regulated apoptosis	[112]

**Table 5:** The anticancer effects of thymoquinone on different cell lines.

Cancer type	Cell line	Effects	Reference
Bladder	HTB-9	Attenuation of PI3K/AKT pathway, antiproliferation, inhibition of epithelial-mesenchymal transition	[118]

Breast	MCF-7	Induced phosphorylation of MAPK and p38, ROS production, antiproliferation, apoptosis,	[119]
	MDA-MB-231	Induced phosphorylation of p38, antitumour in vivo	[119]
	MDA-MB-468, T-47D cells	G1 phase arrest, upregulation of Bax, downregulation of surviving, apoptosis, inhibition of Akt	[120]
	BT549	Reduction of TWIST1 expression, anti-invasion, antimetastasis	[121]
Cholangiocarcinomas	TFK-1, HuCCT1	G2/M arrest, inactivation of PI3K/Akt and NF-ĸB pathways	[122]

Colon	Caco-2, HCT-116, LoVo, HT-29	Antiproliferation	[123]
	DLD-1	Antiproliferation, apoptosis, phosphorylation of MAPK, ERK, JNK, and p38	[123]
Glioblastoma	U-87, CCF-STTG1	Cytotoxicity, antimigration, anti-invasion, antiadhesion, reduction of FAK phosphorylation and ERK expression	[124]
Lung	A549	Reduction of ERK phosphorylation, antiproliferation, antimigration, anti-invasion, inhibition of p16, MMP2, MMP9	[125]

**Table 6:** The anticancer effects of ursolic acid on different cell lines.

Cancer type	Cell line	Effects	Reference
Breast	MCF-7 and MDA-MB-231	Downregulation of STAT3, EFGR, cyclin D1, antiproliferation, cell cycle arrest, apoptosis	[132]
	MDA-MB-231	Regulation of JNK, Akt and mTOR pathways, antimigration, anti-invasion	[133]
	MMTV-Wnt-1	Modulation of Akt/mTOR pathway, apoptosis, cell cycle arrest, antitumour	[134]

Cervical	HeLa	Modulation of p53/MMP-9/PTEN/CD44-mediated signalling pathways, antimetastasis	[135]
Fibrosarcoma	HT1080	Downregulation of MMP-9, antimetastasis	[136]
Gastric	SGC7901	Inhibition of COX-2, cytotoxicity	[64]
		Activation of ROCK1 and PTEN, translocation of cofilin-1, release of cytochrome c, activation of caspase-3 and 9, apoptosis	[137]
	BGC-803	Activation of caspase-3, 8, 9, downregulation of Bcl-2, cell cycle arrest at G0/G1 stage, apoptosis	[82]
	BGC-823	Mitochondrial translocation of cofilin-1, apoptosis	[138]

Glioma	C6	Suppression of ZIP/p62 and PKC-zeta association, downregulation of MMP-9, anti-invasion	[139]
Liver	HepG2	Downregulation of COX-2, antiproliferation, apoptosis	[140]
		Downregulation of survivin, activation of caspase-3, apoptosis	[141]

**Table 7:** Different toxicity studies of the plant extracts on animal models which are under this review.

Plant extract	Animal model	Type of assessment	Route of administration	Behavioral changes	Hematological changes	Biochemical changes	Histopathological changes	References
*Artemisia annua*	Swiss albino mice	Acute toxicity	Oral	No lethality or toxic reaction	Not assessed	Not assessed	Not assessed	[142]

Extract of *Coptidis Rhizoma*	Rats + Kun-ming mice	Acute and subchronic toxicity	Oral	No treatment-related signs of toxicity or mortality	Analysis showed that haemoglobin, red blood cell count (RBC), white blood cell count (WBC), lymph leukocyte count, mononuclea were not significantly affected	Elevation in ALT and AST	Degeneration of hepatocytes in the liver and aggregation of inflammatory cells in the lung	[143]

Extract of *Platycodon grandiflorus*	Sprague–Dawley rats	Subchronic toxicity	Oral	No treatment-related effects on clinical signs, body weight, food and water consumption	No significant differences were observed	Increase in creatinine	Hypertrophy in the liver, and diffuse follicular cell hypertrophy in the thyroid gland	[65]

Extract of *Morus alba* leaves	Swiss mice	Acute toxicity	Oral	No mortality and behavioral alterations	Extract affected MCV, MCHC and leukocytes	Reduction in ALP	Effect on kidneys, liver and spleen	[144]

Extract of*Aristolochia. baetica*	Swiss albino mice	Acute and subacute toxicity	Oral	No mortalities or signs of toxicity. At high dose, caused shortness of breath, abnormal locomotion, and deaths	Increase in creatinine concentration	Significant increase of AST	Renal necrosis, inflammatory infiltrate, and tubular degeneration in kidney organ	[16]

Extract of *Fagonia indica*	Albino mice	Acute toxicity	Oral	No morbidity or behavioral changes	Not assessed	Elevation on both the ALT and AST	Hepatocytes maintained its architecture with normal glycogen storage	[145]

*Thymus vulgaris* L. essential oil	Albino Holtzman rats	Acute and repeated 28-day oral dose toxicity	Oral	Body weight was only altered in male	No significant changes to any of the parameters in the treated groups when compared with the control group	No significant changes	Lungs showed a moderate inflammatory infiltrate. Foci were also observed in rats. Stomach showed a mild acute inflammatory infiltrate	[146]

**Table 8:** Different toxicity studies of the phytochemicals on animal models which are under this review.

Compound	Animal model	Type of assessment	Administration route	Behavioral changes	Hematological changes	Biochemical changes	Histopathological changes	References
1′-S-1′- acetoxychavicol acetate	Sprague-Dawley rats	Acute and 28-day subacute toxicity	Intravenous	No lethality or behavioural changes	All the haematological parameters, were within normal ranges	Significant increase in total protein, albumin and globulin	Mild focal inflammation of kidneys and lobular hepatitis	[148]
Genistein	Wistar rats	Acute, subchronic, and chronic toxicity	Oral	Slightly decreased food consumption and body weight at the highest doses	Decrease in RBCs at the high doses with an increase in reticulocytes.	A slight increase in gamma glutamyl transferase at the high doses	No treatment-related histopathological changes in these studies	[149]
Genistein	Swiss albino mice	Acute toxicity	Intraperitoneal	Not assessed	Not assessed	Elevated ALT, AST, and ALP levels	Degenerated liver tissue and hepatotoxicity	[150]
Thymoquinone-loaded nanostructured lipid carrier	Sprague Dawley rats	Acute toxicity	Intravenous	No significant changes in body weight, food intake.	No changes were reported	No significant differences in ALP, ALT, creatinine, urea, total protein, albumin and total bilirubin	Sec-tions of kidneys and liver showed no abnormality/alterations	[151]
Thymoquinone	Swiss albino mice	Acute and sub-chronic toxicity study	Oral	Hypoactivity and difficulty in respiration at high doses	Increase in urea and creatinine. Significant decrease in fasting plasma glucose level	Increase in ALT, lactate dehydrogenase, and creatine phosphokinase	Significant reduction in tissue (liver, kidneys, and heart)	[152]
Ursolic acid	Han–Wistar rats	Repeated dose (90 days) toxicity	Oral	No toxicological changes were observed	Platelet count was significantly increased in comparison with the control. No other changes were observed	No changes	No changes	[153]
Ursolic acid	Swiss mice	28-day toxicity	Oral	No changes	Ursolic acid revealed elevated neutrophil count. Urea elevation	Not assessed	Alterations in the architecture of the liver, kidney, and spleen tissues	[154]

## Data Availability

Data are available on request.
